# Duodenal Xanthoma: From Specks to Obstruction

**DOI:** 10.7759/cureus.4661

**Published:** 2019-05-14

**Authors:** Sindhura Kolli, Dan C Phan, Mel A Ona

**Affiliations:** 1 Internal Medicine, The Brooklyn Hospital Center, New York, USA; 2 Pathology, Convenant Pathology Services, San Francisco, USA; 3 Gastroenterology, Pali Momi Medical Center, Honolulu, USA

**Keywords:** duodenal xanthoma, xanthoma, foamy macrophages

## Abstract

Xanthomas within the gastrointestinal tract occur secondary to a mucosal insult. When enough cells accumulate, their appearance can range from small nodules studding the intestinal mucosa to bandlike infiltrations to pseudotumor-like masses within the intestine with fibrosis and inflammation resembling malignancy. When large enough, they can produce symptoms of obstruction such as vomiting, abdominal pain, distention, and dysmotility. This case demonstrates the epidemiology, clinical presentation, diagnosis, and treatment of duodenal xanthomas.

## Introduction

Xanthomas are commonly limited to the skin and rarely occur within the gastrointestinal tract. They are caused by an exaggerated response to a past mucosal insult, the lipid laden debris consumed by histiocytes that persist as foamy macrophages. Duodenal xanthomas have a wide spectrum of presentation from whitish speckling of mucosa to larger masses within the small intestine causing obstruction [1-2]. This case demonstrates its epidemiology, clinical presentation, diagnosis, and treatment.

## Case presentation

A 75-year-old Japanese female with gastroesophageal reflux disease (GERD), dyslipidemia, hypothyroidism, and osteoporosis, presented with a two-month history of intermittent dysphagia to solid food associated with bloating and unintentional weight loss of seven pounds over two months. The dysphagia was aggravated with the consumption of starchy foods and alleviated with drinking water.

**Figure 1:**
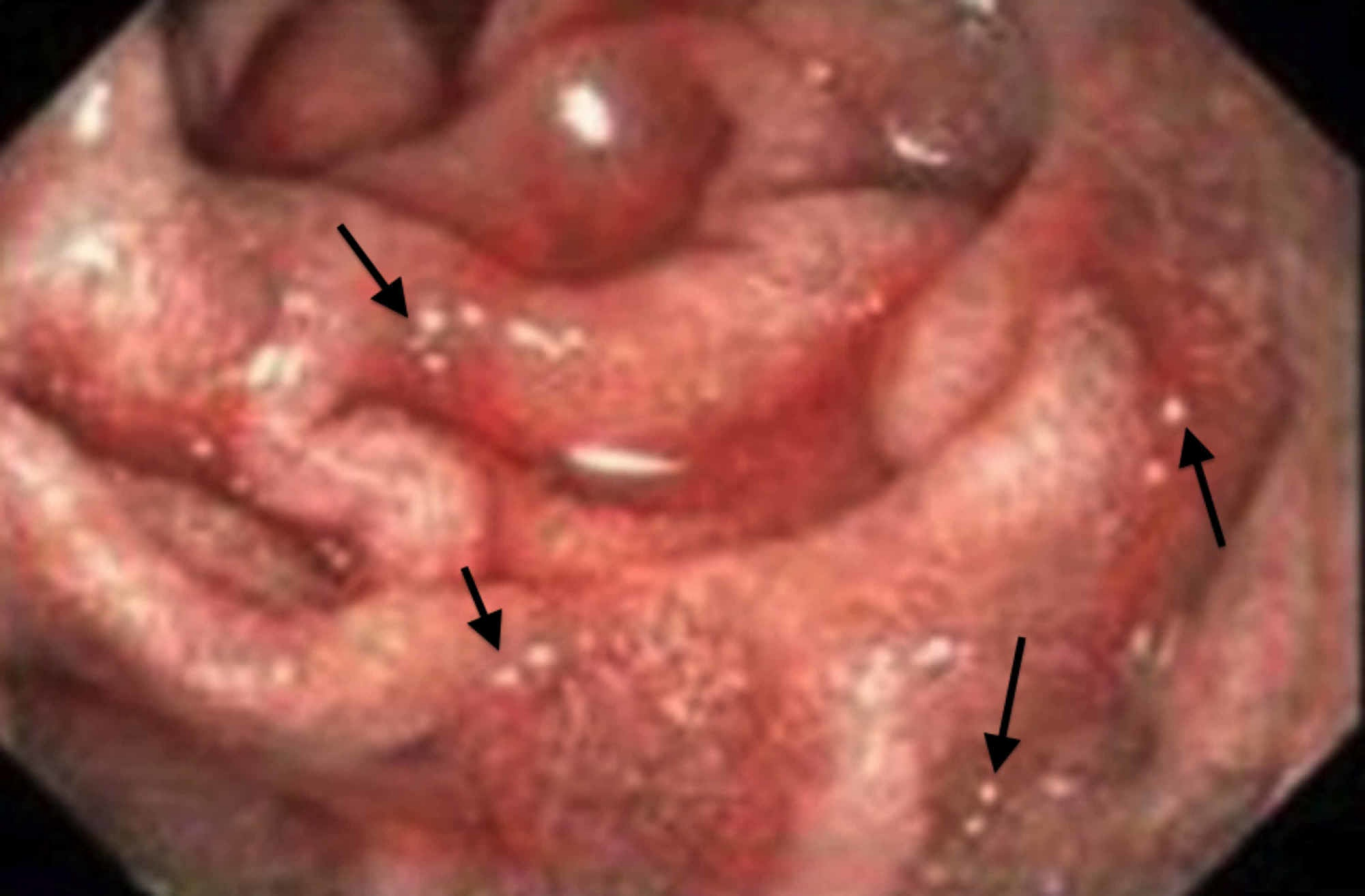
Duodenal xanthoma seen as white speckling of mucosa

**Figure 2:**
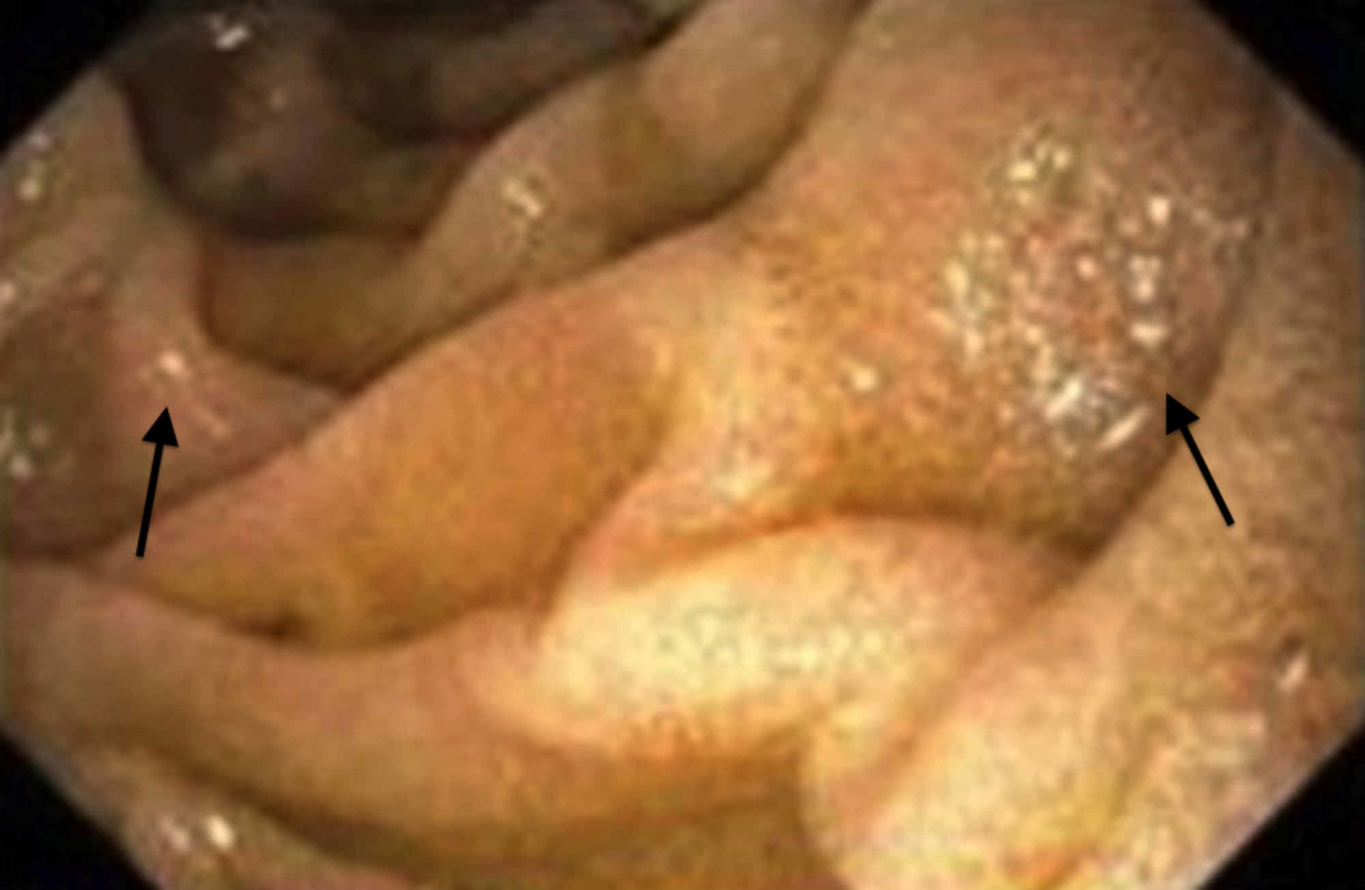
Narrow band imaging of duodenal xanthoma seen as white specks

Esophagram findings demonstrated mild to moderate nonspecific esophageal dysmotility. An esophagoduodenoscopy (EGD) demonstrated gastric erythema and erosions with areas of desquamation in the antrum and prepyloric region (Figures 1-2).

Biopsies were taken from the bulb and second portion of the duodenum which were positive for duodenal xanthoma. The patient was recommended for a repeat procedure for further resection of the xanthoma only if the dysphagia persisted. However, it resolved and the patient was recommended the procedure if the symptoms returned.

## Discussion

In terms of intestinal obstruction, duodenal xanthomas are on the far end of the spectrum of differentials. Xanthomas are considered to be an exaggerated response to a past mucosal insult, the lipid-laden debris consumed by histiocytes that persist as foamy macrophages. With a predilection for skin, they are rarely seen in the gastrointestinal tract. They tend to occur more commonly in the stomach and then in the esophagus (based on literature) with the duodenum as a distant third [1-2]. When enough cells accumulate, their appearance can range from small nodules studding the intestinal mucosa to bandlike infiltrations to pseudotumor-like masses within the intestine with fibrosis and inflammation resembling malignancy. When large enough, they can produce symptoms of obstruction such as vomiting, abdominal pain, distention, and dysmotility [1]. The limited number of cases show a penchant for males 6:1 to females and have a tenuous association with dyslipidemias, chemotherapy, radiation, and cytomegalovirus (CMV) colitis; however, larger studies are needed to determine the significance of these causal relationships [2].

**Figure 3:**
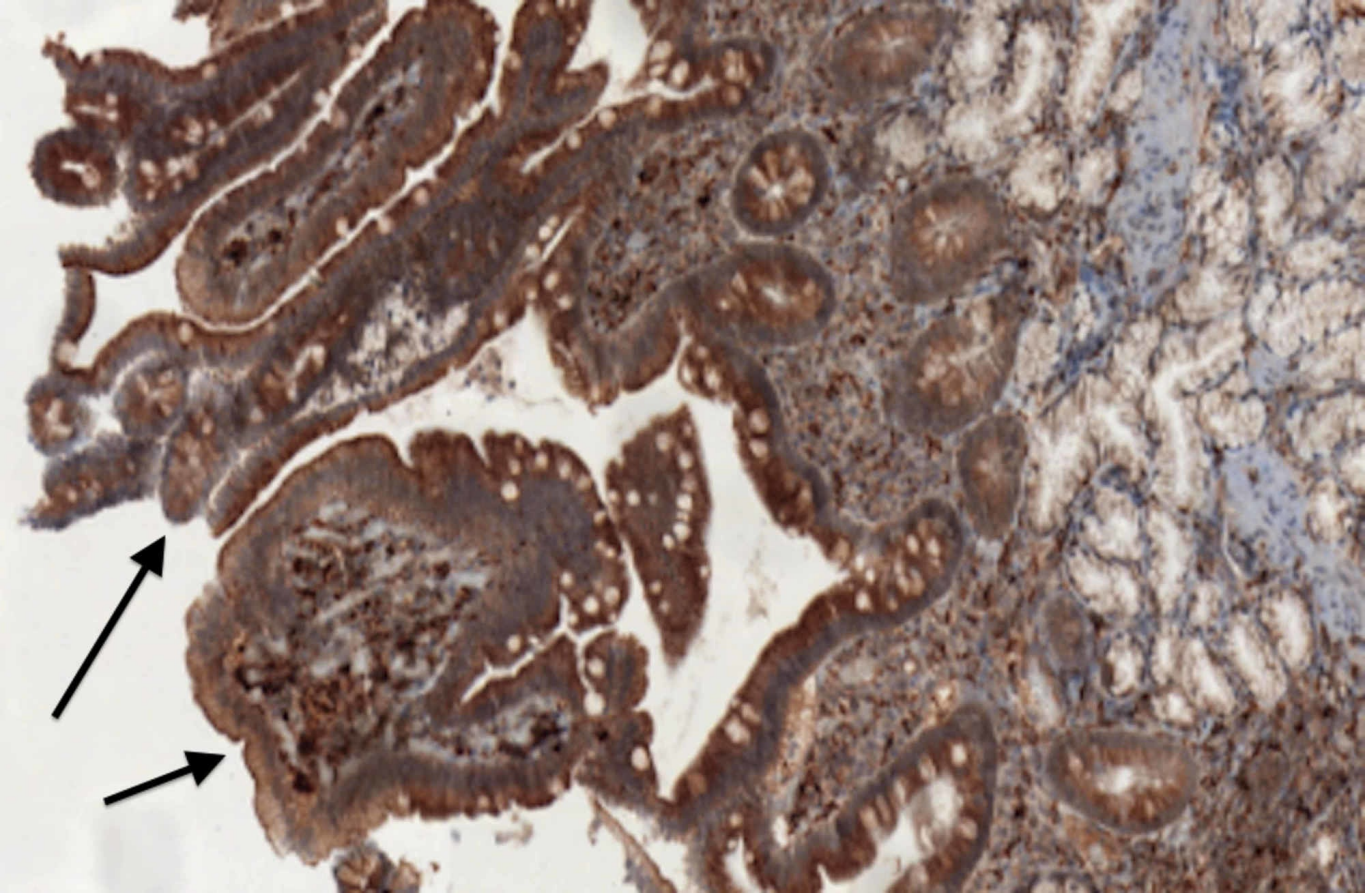
Cluster of differentiation 68 (CD68) positive macrophages on immunohistochemistry (IHC)

Diagnosis is made by endoscopy and biopsy with immunohistochemistry (IHC) staining confirming foamy histiocytes distributed through three layers (serosa, muscularis propria, and submucosa), positive for cluster of differentiation 68 (CD68), decreased smooth muscle, and occasionally accompanied by fibrosis (Figure 3).

**Figure 4:**
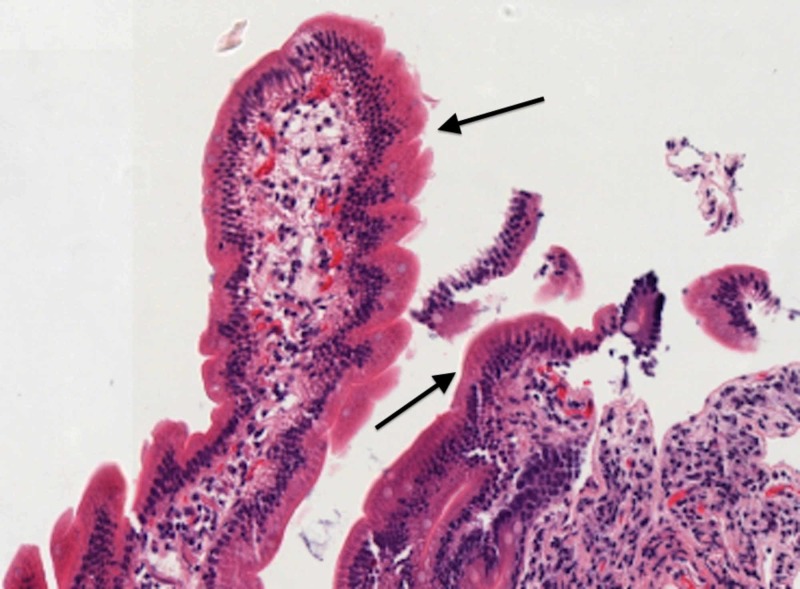
Electron microscopy of foamy macrophages of duodenal xanthoma

Electron microscopy demonstrated foam cells with fatty cytoplasm, as seen in Figure 4 [1-2]. Treatment involves endoscopic mucosal resection and for larger lesions, duodenal preserving surgery [3].

## Conclusions

Despite being an uncommon differential in intestinal obstruction, Xanthomas should be considered when patients with insults from chemotherapy, radiation, or/and CMV colitis present. Xanthomas range in presentation and size, sometimes requiring surveillance, but may also require intestine-preserving surgery. Due to this rare occurrence and wide range of presentation, duodenal xanthomas are not a dismissive matter, rather they require endoscopists' attention.
